# Vitiligo-Like Depigmentation in Nivolumab-Treated Nodular Melanoma: A Paradoxical Clinical Course

**DOI:** 10.7759/cureus.110323

**Published:** 2026-06-05

**Authors:** Frida M Ortega Garcia, Alejandra Gonzalez Uribe, Diana S Blanquet Campos, Martha A Aceves Villalvazo

**Affiliations:** 1 Internal Medicine, Hospital Regional “Dr. Valentín Gómez Farías”, Institute for Social Security and Services for State Workers (ISSSTE), Guadalajara, MEX; 2 Dermatology, Hospital Regional “Dr. Valentín Gómez Farías”, Institute for Social Security and Services for State Workers (ISSSTE), Guadalajara, MEX

**Keywords:** anti-pd-1 therapy, immunotherapy, melanoma, nivolumab, vitiligo-like depigmentation

## Abstract

Vitiligo-like depigmentation (VLD) is a recognized cutaneous adverse event associated with anti-programmed cell death protein-1 (anti-PD-1) therapy in patients with melanoma and has been linked to favorable therapeutic response and improved survival outcomes. We present the case of a 72-year-old woman diagnosed with metastatic nodular melanoma treated with nivolumab monotherapy, who developed disseminated VLD after multiple treatment cycles. Despite achieving an initial complete radiologic response and developing extensive depigmentation, the patient subsequently experienced disease progression, presenting hepatic, pulmonary, and central nervous system metastases. This case contrasts with the commonly reported association between VLD and favorable prognosis, emphasizing that this phenomenon may not consistently predict sustained clinical benefit in patients receiving immunotherapy for advanced melanoma.

## Introduction

Melanoma is a malignant cutaneous neoplasm derived from melanocytes located in the basal layer of the epidermis. In Mexico, it ranks as the 21st most common malignant neoplasm, with approximately 2,051 new cases diagnosed annually. In the Hispanic population, the reported incidence is close to 1.3%, with a peak presentation between 55 and 65 years of age. In recent decades, a significant increase in melanoma-associated mortality has been observed, reaching up to 78% [1,2].

Nodular melanoma is the second most common subtype, accounting for up to 13% of invasive melanomas. Clinically, it is characterized by a rapidly growing exophytic lesion, often friable and ulcerated, with predominant vertical growth. Compared with other melanoma subtypes, nodular melanoma contributes disproportionately to melanoma-related mortality, with a reported five-year survival rate ranging from 38% to 82% [3].

At the time of diagnosis, only a limited proportion of invasive melanomas present with metastatic dissemination, with nodal involvement observed in approximately 9.5% of cases and distant metastases in up to 4.7% [2]. The most commonly affected sites include the lungs, central nervous system, liver, and bone, with reported incidences of 30%, 9.6%, 8.3%, and 5.4%, respectively [4]. Distant metastatic melanoma carries a poor prognosis, with a median survival of 6-9 months in untreated patients and a five-year survival rate of approximately 14% [5,6].

Regarding the management of cutaneous melanoma, tumor thickness and advanced stage carry important therapeutic implications. Surgical excision with 1-2 cm margins remains the primary treatment modality [3]. In advanced-stage and metastatic disease, programmed cell death protein-1 (PD-1) inhibitors such as pembrolizumab or nivolumab, administered either as monotherapy or in combination with ipilimumab as first-line treatment, have demonstrated significant improvements in overall survival. These agents act by blocking the interaction between PD-1 expressed on activated T-cells and its ligands, particularly PD-L1, on tumor cells, thereby preventing T-cell inhibition and restoring antitumor immune responses. By disrupting this immune checkpoint pathway, previously suppressed tumor-specific cytotoxic T-cells are able to proliferate, produce cytokines, and exert antitumor activity. In particular, nivolumab has demonstrated one-year survival rates of up to 70.5% [1,5].

Among the adverse events associated with nivolumab therapy in melanoma patients, vitiligo-like depigmentation (VLD) has been reported, with an estimated incidence of approximately 7.5% [7]. These manifestations usually develop months after treatment initiation and have been associated with antitumor immune responses. However, this relationship is not consistent across all cases, and some patients do not experience a corresponding clinical benefit, leaving the prognostic value of VLD under debate [8].

## Case presentation

We present the case of a 72-year-old woman with a history of type 2 diabetes mellitus and no family history of malignant neoplasms. Her condition began in 2019 with the appearance of an asymptomatic scalp nodule, for which she did not initially seek medical attention.

In June 2021, the patient was evaluated by the dermatology department at a secondary-care hospital due to a 15 mm verrucous lesion on the scalp with a two-year history of progression. She was referred to the surgical department, where an excisional biopsy was performed. Histopathologic examination revealed ulcerated nodular melanoma with a Breslow thickness of 5 mm, Clark level V, and negative surgical margins. As part of the staging workup, computed tomography (CT) scans of the brain, chest, abdomen, and pelvis were obtained, revealing a posterior cervical lymph node, mediastinal lymphadenopathy, and left pulmonary nodules suggestive of metastatic disease.

Subsequently, in August 2021, she was referred to our tertiary-care center for evaluation by the oncology department. Further workup was completed, and treatment with nivolumab at a dose of 280 mg every three weeks was initiated. Additionally, the surgical oncology team planned an excisional biopsy of the cervical lymph node; however, the patient was lost to follow-up before the procedure could be performed.

After four months of immunotherapy, follow-up CT showed no evidence of active disease. Beginning with cycle 11, approximately nine months after treatment onset, the patient developed areas of depigmentation on the face and extremities. She was subsequently referred to dermatology, where physical examination revealed a disseminated dermatosis involving the head, trunk, and upper and lower extremities, including the supraciliary, perioral, and malar regions, as well as the periumbilical area, dorsal aspect of both arms, flanks, and the anterior and medial thighs. The lesions consisted of confluent hypochromic macules of varying sizes, with smooth surfaces and well-defined borders (Figure 1). Based on the clinical findings, nivolumab-induced VLD was diagnosed. Photoprotection measures and skin care recommendations were indicated, and immunotherapy was continued, as this phenomenon was considered a marker of therapeutic response.

**Figure 1:**
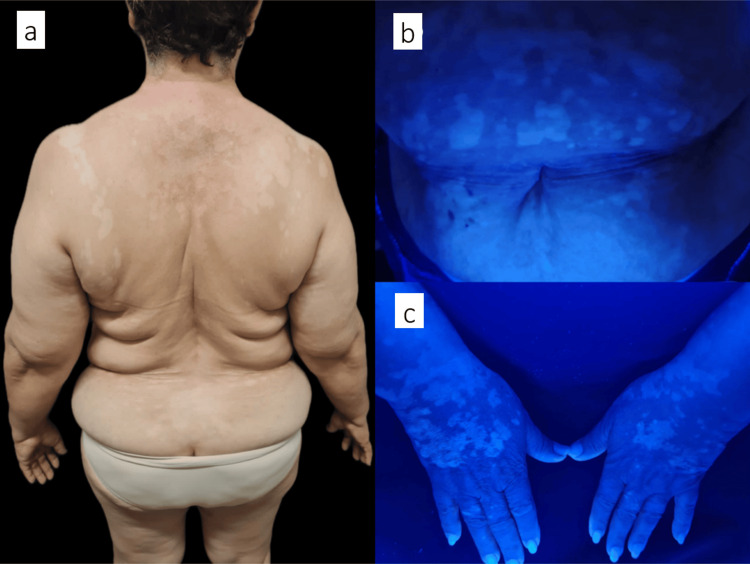
Vitiligo-like depigmentation. (a) Disseminated dermatosis involving the trunk and upper extremities, consisting of confluent hypochromic macules of varying sizes, with smooth surfaces and well-defined borders. (b, c) Accentuation of the depigmented lesions under Wood’s lamp examination.

In April 2024, after completing 34 cycles of nivolumab, new imaging studies (brain, chest, abdomen, and pelvic CT scans) showed no evidence of new metastatic lesions (Figures 2a, 2b). Therefore, nivolumab was discontinued, and quarterly surveillance was continued. After seven months of progression-free survival, a liver lesion was identified on imaging studies in October 2024, and malignancy was confirmed histopathologically. Consequently, nivolumab rechallenge therapy was initiated, concurrently documenting progression of the VLD. In August 2025, after completing the sixth cycle of nivolumab rechallenge, metastatic activity involving the lungs, liver, and central nervous system was documented (Figures 2c, 2d).

**Figure 2:**
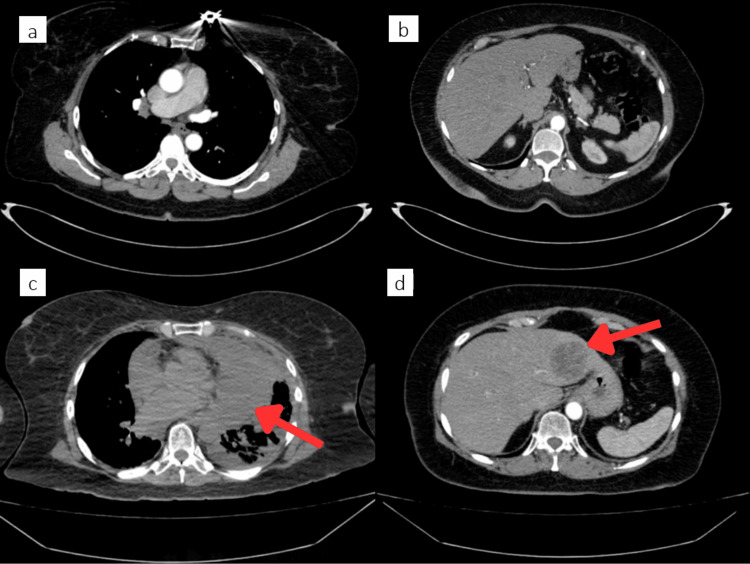
Follow-up computed tomography (CT) scans demonstrating disease evolution. (a, b) April 2024: no radiologic evidence of progression after 34 cycles of nivolumab. (c, d) August 2025: diffuse metastatic progression involving lungs and liver.

Due to disease progression, a single cycle of dacarbazine chemotherapy was administered, and the patient was later transitioned to palliative care. The patient’s clinical evolution, radiologic findings, and treatment course are summarized in Table 1.

**Table 1:** Summary of the patient’s clinical course and treatment. CT: computed tomography; VLD: vitiligo-like depigmentation

Date	TNM	Stage	Clinical findings	Treatment
June 2021	T4bNxMx	III	Ulcerated 15 mm scalp nodule. Histopathology confirmed nodular melanoma.	Complete surgical excision
August 2021	T4bN2bM1b	IV	CT demonstrated cervical and mediastinal lymphadenopathy and pulmonary nodules suggestive of metastatic disease.	Nivolumab monotherapy initiated
January 2022		IV	Radiologic resolution of pulmonary nodules.	Cycle 8 complete response to immunotherapy
May 2022		IV	Development of vitiligo-like lesions.	Cycle 11 continued immunotherapy
April 2024		IV	Extension of VLD without radiologic evidence of disease progression.	Completed 34 cycles of nivolumab
November 2024	T4bN2bM1c	IV	CT revealed a hepatic lesion suggestive of tumor activity.	Liver biopsy performed
December 2024		IV	Liver biopsy confirmed metastatic melanoma.	Rechallenge therapy with 6 cycles of nivolumab plus 1 cycle of dacarbazine
August 2025	T4bN3bM1d	IV	Metastatic progression involving lungs, liver, and central nervous system.	Transitioned to palliative care

## Discussion

Nivolumab is a fully human immunoglobulin G4 monoclonal antibody directed against the PD-1 receptor. By inhibiting PD-1 signaling, nivolumab enhances T-cell-mediated antitumor immune responses [5]. In 2015, the European Medicines Agency approved nivolumab for the treatment of metastatic melanoma, representing a major advance in the prognosis of these patients, significantly improving overall survival and progression-free survival, which may reach up to 22 months in stage III-IV disease, even as monotherapy. PD-1 expression is present in approximately 40%-50% of melanoma patients [9-13].

Together, anti-PD-1 immunotherapy and targeted therapy with BRAF and MEK inhibitors have substantially transformed the management of advanced melanoma. Nevertheless, these therapeutic strategies are associated with a broad spectrum of cutaneous adverse events, including maculopapular eruptions, pruritus, xerosis, photosensitivity, keratoses, and pigmentary alterations such as VLD [9].

The pathogenesis of VLD remains incompletely understood. Several mechanisms have been proposed, primarily involving cross-reactivity against antigens shared by melanoma cells and melanocytes (GP100, MART-1, TRP1, TRP2, and tyrosinase), leading to immune-mediated melanocyte destruction and the subsequent development of vitiligo-like lesions, clinically manifested as depigmented areas. Cases of skin repigmentation coinciding with tumor recurrence have also been described, suggesting a dynamic relationship between immune activity and tumor burden [13-15].

VLD associated with immunotherapy typically develops late after multiple treatment cycles, although the onset time is variable. According to Ramondetta et al., the average onset of VLD occurred 6.9 months after treatment initiation [7,9]. Clinically, VLD presents as bilateral achromic macules with a tendency toward symmetry. Unlike classic vitiligo, it may exhibit atypical patterns and appears to have less predilection for acral areas [7].

Several cohort studies and meta-analyses have suggested that the development of VLD during PD-1 inhibitor therapy may correlate with favorable therapeutic outcomes. In particular, the study by Teulings et al., which included 27 studies, demonstrated a significant association between VLD and improved progression-free and overall survival [8]. Similarly, in a cohort study of 148 patients treated between 2007 and 2017, VLD was more frequently associated with PD-1 inhibitor therapy, occurring in 56% of cases [16,17].

## Conclusions

VLD is a recognized cutaneous adverse event associated with anti-PD-1 therapy, particularly in patients with melanoma, and has been correlated with favorable outcomes in several studies. However, the present case contrasts with the existing literature, as VLD development has generally been associated with improved prognosis and prolonged progression-free survival, whereas our patient experienced progression of metastatic disease.

This finding highlights the heterogeneity of responses to immunotherapy and suggests that, although VLD may represent an immunologic marker of antitumor activation, it should not be considered an absolute predictor of favorable clinical response. Therefore, this phenomenon must be interpreted within the broader clinical context of each individual patient.
